# Relationship Between Obstructive Sleep Apnea and Sports—Systematic Review and Meta-Analysis

**DOI:** 10.3390/jcm13226814

**Published:** 2024-11-13

**Authors:** Lucía Martínez Revuelta, Javier Flores-Fraile, Álvaro Zubizarreta-Macho, José María Montiel-Company, Ana Belén Lobo-Galindo, Patricia Arrieta Blanco

**Affiliations:** 1 Faculty of Dentistry, Alfonso X El Sabio University, 28691 Madrid, Spainamacho@uax.es (Á.Z.-M.); parribla@uax.es (P.A.B.); 2 Department of Surgery, Faculty of Medicine and Dentistry, University of Salamanca, 37008 Salamanca, Spain; 3 Department of Stomatology, Faculty of Medicine and Dentistry, University of Valencia, 46010 Valencia, Spain; jose.maria.montiel@uv.es

**Keywords:** obstructive sleep apnea, sports, exercise, physical activity, fitness

## Abstract

The primary objective of this research is to analyze and compare the effects of physical activity on patients diagnosed with obstructive sleep apnea (OSA), a condition characterized by repeated episodes of partial or complete obstruction of the upper airway during sleep, leading to disrupted sleep and various health complications. **Methods:** A systematic and strategic search of 16 peer-reviewed articles was conducted to assess the impact of different types of physical exercise on the apnea–hypopnea index (AHI), a key metric used to quantify the severity of OSA. The selected studies focused on two main exercise modalities: aerobic exercise alone and a combination of aerobic and resistance training. These modalities were examined to determine their respective influences on the AHI among individuals with OSA. **Results:** The findings revealed that aerobic exercise alone did not significantly alter the apnea–hypopnea index (*p* = 0.15), suggesting limited effectiveness in reducing the severity of OSA when performed in isolation. However, in patients who engaged in combined physical activity—incorporating both aerobic and resistance exercises—a significant reduction in the AHI was observed (*p* < 0.01), indicating a more substantial therapeutic effect. **Conclusions:** This study concludes that a structured exercise program combining aerobic and resistance training produces a significant improvement in the apnea–hypopnea indices of patients with obstructive sleep apnea. These results highlight the potential of combined exercise regimens as a non-pharmacological intervention that could complement traditional OSA treatments, offering a viable approach to managing the condition and improving patient outcomes. Future research should aim to explore the optimal exercise intensity, duration, and frequency to maximize these benefits.

## 1. Introduction

Obstructive sleep apnea (OSA) is a slowly progressive pathophysiological condition of partial or total upper airway obstruction produced by a repeated series of collapses of the oropharynx (back position of the throat) during rest that intermittently last for more than ten seconds. This can occur with or without oxygen saturation, i.e., there may be an impediment to the body receiving sufficient doses of oxygen. The number of apneas can be determined by means of the apnea–hypopnea index (AHI), which is obtained by dividing the total number of recorded apneas by the minutes of sleep and then multiplying by 60 [1,2,3,4].

Using this value and its relation with the values estimated by the “American Academy of Sleep Medicine”, sleep apneas can be qualified as non-pathological or pathological, which is characterized by greater than five apneas per sleep session and is classified as mild (5–15), moderate (15–30), or severe (greater than 30) [4].

There are three types of apneas:

- Obstructive apneas are the most frequent and are produced by the closure of the airways, impeding airflow and making ventilatory effort necessary. This cessation is maintained for more than ten seconds. We have also previously mentioned the possibility of a reduction in oxygen flow which, together with the interruption of sleep during the night, gives rise to a subgroup of obstructive apneas called hypopneas, which are collectively referred to as obstructive sleep apnea hypopnea syndrome (OSAHS). These are characterized by a ventilatory flow rate that is half of the normal value [1,5].
- Central apneas occur when the respiratory muscles present a temporary cancellation of a central stimulus. On the one hand, like obstructive apneas, the duration of respiratory arrest is above ten seconds, and there is a need for ventilatory effort. On the other hand, what differentiates central from obstructive apneas is that the micro awakenings are caused by the complete lack of ventilatory flow in the former [1,5].
- Mixed type apneas are characterized by central apnea primary to an obstruction [5].

Obstructive sleep apneas are physiologically characterized by a narrow and collapsible upper airway. During the time the patient is awake, the airway remains patent, i.e., has a normal width, as a consequence of the action of the airway dilator muscles. The action of these muscles decreases; therefore, the equilibrium is broken during sleep, leading to collapse. At the neurological level, what occurs is the joint action of neurotransmitters, in which serotonin plays a major role in stimulating a motor neuronal excitation of the upper airway. Therefore, an alteration in these signals causes obstruction of the airways and a consequent effect on obstructive sleep apnea [1,5].

Despite being an underdiagnosed and underreported disease, it has a high prevalence in people with risk factors and associated diseases, such as obesity (which is the focus of our work), sex, age, respiratory failure, coronary artery disease, heart failure, hypothyroidism, and acromegaly [2,5,6,7].

Continuous positive airway pressure (CPAP) has so far been the treatment of choice for obstructive sleep apnea. Over time, research and increased knowledge about this disease has allowed for the implementation of reinforcement techniques in orthodontics such as mandibular advancement devices and maxillary bone widening, and the incorporation of new habits such as physical activity and a healthy diet [7,8,9].

As mentioned above, obesity is a condition that can be modified by the aforementioned resources and is a major risk factor for the development of OSA and consequently AHI, and reversing it can improve the quality of sleep [10,11].

It has been found that physically active people are less likely to be obese. There is no clear evidence as to which type of physical activity, i.e., aerobic or strength training, is more effective. The effect of training on the OSA patient is a noticeable weight loss combined with general toning of the body muscles, creating a greater resistance of the ventilatory muscles and upper airway dilators to fatigue; these effects help regulate sleep balance and reduce nasal blockage. Independently of what has been described above, other results have been found [10,11,12,13].

Previous studies have shown an association between obesity [2,5,6,7] and the risk of suffering from OSA and have highlighted the beneficial effect of practicing sports to prevent this pathology; however, it is not known which exercise is the most beneficial: aerobic exercise or a combination of aerobic and resistance exercise. This study was designed to provide a recommendation for overweight people affected by OSA.

It has been observed that patients with OSA do not perform physical exercise, and the presence of this disease is lower in those who regularly practice physical exercise [13,14,15]. Therefore, the aim of this study was to determine the relationship between obstructive sleep apnea and sports by analyzing the effect of the practice of sports on health changes and improvements in OSA, and the null hypothesis (H_0_) is that there is no difference between obstructive sleep apnea and the practice of sports in improvements in this pathology.

## 2. Materials and Methods

### Study Design

The literature review was conducted following guidelines for systematic reviews and meta-analyses in accordance with PRISMA (Preferred Reporting Items for Systemic Reviews and Meta-Analyses, http://www.prisma-statement.org (accessed on 14 February 2024); International Prospective Register of Systematic Reviews (INPLASY) registration number: CRD42020192179). The review was also complied with the PRISMA 2009 Checklist [14] and was performed in accordance with current recommendations regarding systematic reviews and meta-analyses. The population, intervention, comparison, and outcome (PICO) question was “Is there any relationship between obstructive sleep apnea and sport? With the following components: population: patients with obstructive sleep apnea and practicing sport; intervention: aerobic exercise; comparison: apnea–hypopnea index (AHI) outcome: improvement or not of the apnea–hypopnea index (AHI)”.

An electronic search was carried out using the following databases: PubMed, Scopus, Cochrane, and Web of Sciences. The search assessed all the literature published internationally up to March 2024. Five medical subject heading (MeSH) terms were included in the search: “obstructive sleep apnea”; “sports”; “exercise”; “physical activity”; and “fitness”. One Boolean operator was applied (“AND”). These search terms were applied as follows: [(“obstructive sleep apnea”) AND (“sports”) AND (“exercise”) AND (“physical activity”) AND (“fitness”)]. Two different researchers (S.D. and A.Z.M.) searched the databases simultaneously. The inclusion and exclusion criteria were applied to titles, and a single researcher (S.D.) extracted the data regarding the relevant variables. A.Z.M. conducted the systematic review, and two researchers who had not participated in the selection process (A.Z.M. and S.D.) performed the subsequent meta-analysis.

The inclusion criteria were as follows: studies registered in impact journals and databases such as prospective and retrospective randomized clinical trials (RCT) with a small time range that referred to obstructive sleep apnea and its relationship with sports (last ten years) and with a somewhat larger range to contrast the changes in this condition, as well as diagnostic methods and related aspects. In addition, the search for articles was carried out in both Spanish and English, the latter having a greater weight due to the greater number of studies and information that exists in this language. Studies were not restricted by language or year of publication. The exclusion criteria were as follows: systematic literature reviews, clinical cases, case series, and editorials. The following data were recorded: author, year, title, journal, and sample size (n). The results were obtained from studies that analyzed the relationship between physical exercise and improvement in OSA patients.

The risk of bias of the clinical studies selected for review was assessed using the Jadad scale for methodological quality assessment of clinical trials. The Jadad scale consists of five items that evaluate randomization, researcher and patient blinding, and description of losses during follow-up, producing a score of 0–5; scores of less than 3 are considered low quality [15].

The meta-analysis by subgroup (aerobic exercise and aerobic exercise + resistance) was carried out using the random effects model and inverse variance method. The effect size was measured as the difference in means of the apnea–hypopnea index of the two experimental groups that engaged in physical exercise compared to the control group. The significance of the meta-analysis was assessed using the z test in which *p* < 0.05. Heterogeneity was analyzed using the I2, and the difference between the subgroups was assessed using the Q test. The meta-analysis was represented using a forest plot.

Publication bias was analyzed using the trim-and-fill method of funnel plot skew adjustment.

## 3. Results

### 3.1. Flow Diagram

**Figure 1:**
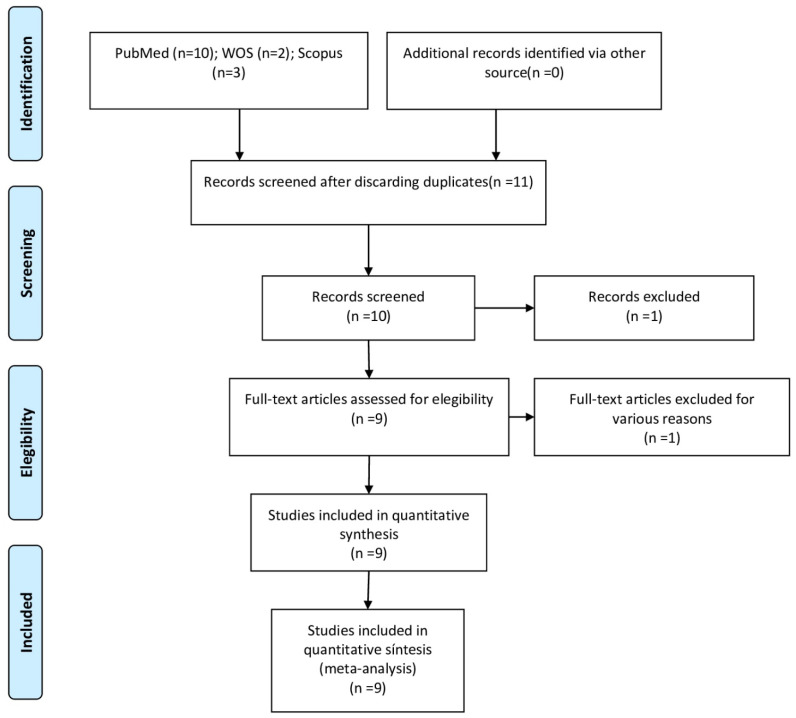
Preferred Reporting Items for Systematic Reviews and Meta-Analyses (PRISMA) flow diagram.

A total of 31 articles in PubMed, 17 articles in Web of Sciences, and 2 articles in Scopus were found after the initial search. Of these 50 works, 6 duplicates were discarded. After assessing study titles and abstracts, another 8 were eliminated, after which 36 remained. An additional 16 were rejected for failing to fulfill the inclusion criteria by either not including the relationship between obstructive sleep apnea and sports or articles whose subjects are syndromic patients. After this selection process, a total of 16 articles were selected for the final qualitative synthesis. Sixteen articles were ultimately assessed in the quantitative analysis, as they met all the selection criteria (Figure 1).

### 3.2. Qualitative Analysis

All of the 16 articles that were included were randomized clinical trials (CT) (Table 1). All studies except for two had a sample size greater than 10, which was the smallest value in the study by Sengul et al. (2011) [9] and the highest value in the study by Ueno-Pardi et al. (2022) [16].

### 3.3. Quality Assessment

Table 2 shows the results of the methodological quality assessment using the Jadad scale. All the articles selected for this meta-analysis [9,10,11,12,13,16,17,18,19,20,21,22,23,24,25,26] presented two points on the Jadad scale, giving rise to a deficient character due to the quality of the methodology. This score was obtained only for the “Allocation Sequence and Randomization Process” and “Statistical Analysis”, thus compromising the fulfillment of the criteria linked to the randomization and masking processes.

### 3.4. Quantitative Analysis

The meta-analysis of the experimental aerobic exercise subgroup revealed no significant effect (*p* = 0.15). A total of 13 studies were combined using a random effects model, which estimated a mean difference of 0.77 (with a 95% confidence interval between −1.16 and 2.70) compared to the control group. The heterogeneity was slight, with I^2^ = 30%.

On the contrary, the meta-analysis of the experimental subgroup of aerobic exercise + resistance revealed a significant mean difference (*p* < 0.01), combining 12 studies and estimating a difference in the apnea–hypopnea index of −5.31 (with a 95% confidence interval between −8.43 and −2.18) between the experimental and control groups. The heterogeneity was high, with I^2^ = 79%.

**Figure 2:**
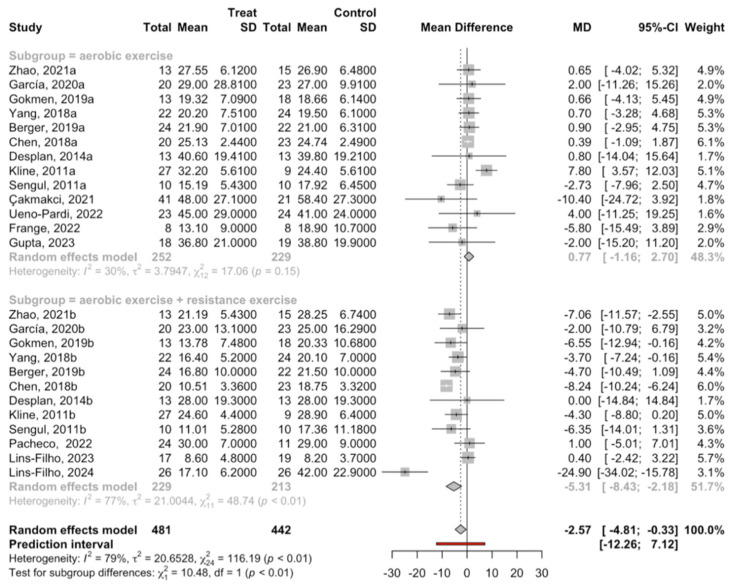
Forest plot of the mean differences in the apnea–hypopnea index (AHI) after meta-analysis by subgroup.

There were significant differences in the effect on AHI between the two subgroups (Q test = 10.5; *p* = 0.001) (Figure 2).

### 3.5. Publication Bias

**Figure 3:**
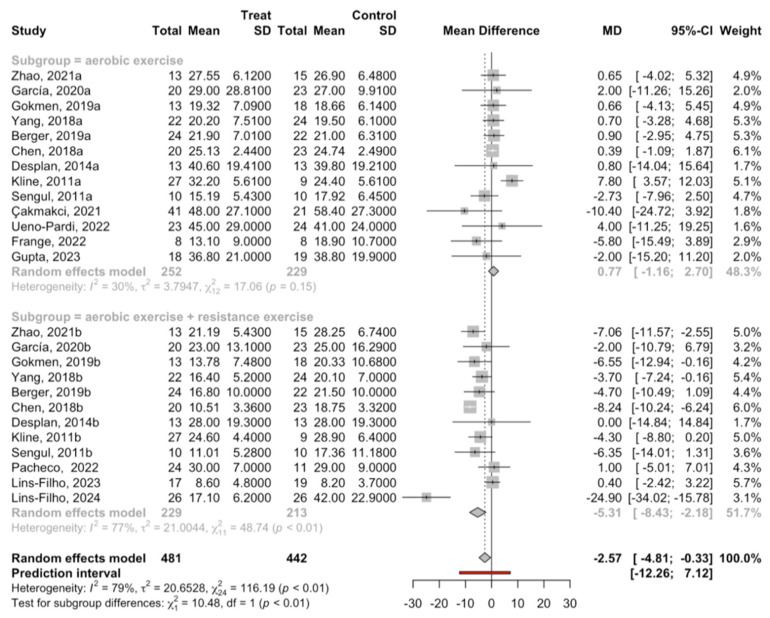
Funnel plots of the mean differences in apnea–hypopnea index using the trim-and-fill method after meta-analysis, with the initial estimate (left funnel plot) and the final estimate (right funnel plot).

Publication bias was studied using the trim-and-fill method of adjusting the asymmetry of the funnel plot, in which no new studies were added, which confirms the absence of bias (Figure 3).

## 4. Discussion

The results obtained in the present study confirm the null hypothesis (H_0_), which holds that there is no difference between obstructive sleep apnea and the practice of sports regarding improvements in patient outcomes related to this pathology.

As we have seen, there is a reciprocal relationship between obstructive sleep apnea and the practice of sports. Despite its recent incorporation into the clinical setting, sports have been studied extensively, but the evidence obtained in available studies and research is not sufficient. This means that although it is not the main subject of this study, there are other factors that jointly influence the results of physical activity and apnea–hypopnea rates, such as muscle mass index, oxygen saturation, heart rate, respiratory pressure, and diet, among others; these five are the most common features included in the studies used for this meta-analysis. The following is an analysis and comparison of the results obtained from studies and bibliographic searches carried out by certain authors.

After the study of several meta-analyses, it was concluded that aerobic exercise combined with resistance in patients with obstructive sleep apnea significantly improved outcomes related to this disease, particularly, the weight loss that is often a result of physical exercise.

Finally, Zhao et al. [16] observed that the combination of aerobic exercise and resistance increases exercise capacity and heart rate in subjects with OSAHS, thus reducing the severity of the disease. Garcia et al. [10], based only on aerobic and home exercise, concluded that this practice favored the lowering of oxygen desaturation and cholesterol indices, contributing in the same way to improvements in OSAHS. With these results, they affirmed that the habitual practice of short bouts of home exercise is a simple treatment for OSA. In the same way, Yang et al. [13] studied subjects who were only engaged in aerobic exercise, and their results were positive, suggesting that such activity leads to a decrease in body mass index and a reversal of AHI. Curiously, in the same line of the previous conclusions of the cited authors, Chen et al. [18] shows a clear relationship between improvements in OSA and aerobic sports together with resistance but assures that this result is independent of the changes in the body mass index, that is to say, physical exercise does not decrease this index, but it does improve OSA. Pacheco et al. [21] achieved the same results. In addition, they showed that although body mass index did not influence OSA, changes in fluid retention could lead to variable effects. Gokmen et al. [12] obtained contradictory results: aerobic exercise at home was much less efficient than programmed training with a professional, and therefore, the results regarding obstructive sleep apnea in the first group were null, while in the second group, there was a significant difference, with an improvement in the quality of sleep and physical condition. In a study by Berger et al. [17], the practice of supervised physical activity was adapted to each person to obtain positive results regarding the rates of apnea and hypopnea, and they observed health improvements in patients who presented with cardiovascular problems. Desplan et al. [19], like Chen et al. [18], looked more closely at body mass index. In their case, the results supported the influence of BMI reductions after regular exercise on improvements in OSA, affirming the existence of a clear relationship between them and therefore leaving open a path of study to classify physical exercise as a treatment for respiratory pathology. The objective of Kline et al. [11] was to evaluate the efficacy of training as a therapeutic measure in apnea independently of the loss or maintenance of body mass. Despite the small sample size, their results showed a correlation between these variables. CPAP and surgical treatment were compared, and the improvement was more notable with the CPAP device than with the surgical intervention. They concluded that sports are a significant mediator in the improvement of apneas. Sengul et al. [9] obtained significant results in their study. Frange et al. [23] also found a correlation between both variables. Çakmakci et al. [20] focused on the introduction of physical exercise as a reinforcement to CPAP treatment and obtained favorable results that indicated that the use of this combination was beneficial. Ueno-Pardi et al. [22] analyzed the alterations in glucose metabolism in patients with OSA. They found that the practice of exercise improved oxygen desaturation and the apnea–hypopnea index, indicating a direct relationship between the two and indirectly improving the consequences of apnea, such as decreasing drowsiness and increasing attention. Gupta et al. [24] suggested that obesity is a risk factor for OSA and attributed it to the increase in OSA prevalence. In this study, we chose to perform aerobic exercises such as yoga to see if there was an improvement in OSA, and satisfactory results were obtained. Hellrigel-Holderbaum et al. [27] found that decreases in weight, lipid levels, drowsiness, and the number of respiratory disorders after physical activity and a restructuring of the diet notably improved AHI. Finally, Lins-Filho et al. [25,26] conducted two different studies over consecutive years; in the first one, they studied the effect of the periodic practice of rigorous high-intensity interval training (HIT) on obstructive sleep apnea, and in the second one, they assessed the influence of a sedentary lifestyle on the presence of OSA. These two studies differ in both duration and number of subjects and showed that training decreases AHI and mitigates OSA, which improved the performance and physical condition of the population that was studied.

In addition, the randomized controlled trials selected in the present systematic review and meta-analysis have low methodological quality; therefore, the authors highlight the necessity of improving the methodological design for future randomized controlled trials related to this topic. Moreover, the authors also highlight the influence of confounding factors not reported in some of the included articles, such as BMI, fluid retention, or other lifestyle modifications, that could have significantly impacted the outcomes. The authors also encourage future researchers to analyze the influence of other important clinical outcomes, such as quality of life, cardiovascular health, and daytime sleepiness, as well as to increase the follow-up periods to evaluate the long-term sustainability of the effects of the intervention on OSA severity.

## 5. Conclusions

One conclusion derived from the present study is that there is a significant difference between the practice of aerobic sports in combination with endurance and improvements in patients with obstructive sleep apnea. On the other hand, the practice of aerobic physical exercise does not produce beneficial changes in patients with this pathological condition.

## Figures and Tables

**Table 1:** Qualitative analysis of articles forming part of the systematic review.

Author (Year)	Study Type	Sample (*n*)		AHI	
		Training	Control	Training	Control
ZHAO et al. (2021) [16]	RCT	20 (13)	20 (15)	27.55 + 6.12	26.90 + 6.48
		20 (13)	20 (15)	21.19 + 5.43	28.25 + 6.74
García et al. (2020) [10]	RCT	34 (20)	34 (23)	29.0 + 20.8	27.0 + 9.9
		34 (20)	34 (23	23.0 + 13.1	25.0 + 16.29
Gokmen et al. (2019) [12]	RCT	25 (13)	25 (18)	19.32 + 7.09	18.66 + 6.14
		25 (13)	25 (18)	13.78 + 7.48	20.33 + 10.68
Yang et al. (2018) [13]	RCT	32 (22)	35 (24)	20.2 + 7.5	19.5 + 6.1
		32 (22)	35 (24)	16.4 + 5.2	20.1 + 7.0
Berger et al. (2019) [17]	RCT	36 (24)	38 (22)	21.9 + 7.0	21.0 + 6.3
		36 (24)	38 (22)	16.8 + 10.0	21.5 + 10.0
Chen et al. (2018) [18]	RCT	35 (20)	35 (23)	25.13 + 2.44	24.74 + 2.49
		35 (20)	35 (23)	10.51 + 3.36	18.75 + 3.32
Desplan et al. (2014) [19]	RCT	13 (13)	13 (13)	40.6 + 19.4	39.8 + 19.2
		13 (13)	13 (13)	28.0 + 19.3	28.0 + 19.3
Kline et al. (2011) [11]	RCT	27 (15)	16 (9)	32.2 + 5.6	24.4 + 5.61
		27 (15)	16 (9)	24.6 + 4.4	28.9 + 6.4
Sengul et al. (2011) [9]	RCT	10 (10)	10 (10)	15.19 + 5.43	17.92 + 6.45
		10 (10)	10 (10)	11.01 + 5.28	17.36 + 11.18
Çakmakci et al. (2021) [20]	RCT	41 (20)	41 (21)	58.4 + 27.0	48.0 + 27.1
Pacheco et al. (2022) [21]	RCT	24 (12)	24 (11)	29.0 + 9.0	30.0 + 7.0
Ueno-Pardi et al. (2022) [22]	RCT	47 (23)	47 (24)	41.0 + 24.0	45.0 + 29.0
Frange et al. (2022) [23]	RCT	20 (8)	20 (8)	18.9 + 10.7	13.1 + 9.0
Gupta et al. (2023) [24]	RCT	37 (18)	37 (19)	38.8 + 19.9	36.8 + 21,0
Lins-Filho et al. (2023) [25]	RCT	28 (17)	38 (19)	8.2 + 3.7	8.6 + 4.8
Lins-Filho et al. (2024) [26]	RCT	26 (16)	26 (26)	42.0 + 22.9	17.1 + 6.2
ET: Controlled Trial.					

**Table 2:** Methodological quality assessment as per the Jadad scale.

Author (Year)	Sample Preparation and Handling	Allocation Sequence and Randomization Process	Whether the Evaluators Were Blinded	Statistical Analysis
ZHAO et al. (2021) [16]	0	1	0	1
García et al. (2020) [10]	0	1	0	1
Gokmen et al. (2019) [12]	0	1	0	1
Yang et al. (2018) [13]	0	1	0	1
Berger et al. (2019) [17]	0	1	0	1
Chen et al. (2018) [18]	0	1	0	1
Desplan et al. (2014) [19]	0	1	0	1
Kline et al. (2011) [11]	0	1	0	1
Sengul et al. (2011) [9]	0	1	0	1
Çakmakci et al. (2021) [20]	0	1	0	1
Pacheco et al. (2022) [21]	0	1	0	1
Ueno-Pardi et al. (2022) [22]	0	1	0	1
Frange et al. (2022) [23]	0	1	0	1
Gupta et al. (2023) [24]	0	1	0	1
Lins-Filho et al. (2023) [25]	0	1	0	1
Lins-Filho et al. (2024) [26]	0	1	0	1

## Data Availability

The datasets generated and/or analyzed during the current study are not publicly available due to the involvement of the personal data of patients, but they are available from the corresponding author on reasonable request.
